# Eco-friendly streamlined process for sporopollenin exine capsule extraction

**DOI:** 10.1038/srep19960

**Published:** 2016-01-28

**Authors:** Raghavendra C. Mundargi, Michael G. Potroz, Jae Hyeon Park, Jeongeun Seo, Ee-Lin Tan, Jae Ho Lee, Nam-Joon Cho

**Affiliations:** 1 School of Materials Science and Engineering, Nanyang Technological University, 50 Nanyang Avenue 639798, Singapore; 2 Centre for Biomimetic Sensor Science Nanyang Technological University 50 Nanyang Drive 637553, Singapore; 3 School of Chemical and Biomedical Engineering Nanyang Technological University 62 Nanyang Drive 637459, Singapore

## Abstract

Sporopollenin exine capsules (SECs) extracted from *Lycopodium clavatum* spores are an attractive biomaterial possessing a highly robust structure suitable for microencapsulation strategies. Despite several decades of research into SEC extraction methods, the protocols commonly used for *L. clavatum* still entail processing with both alkaline and acidolysis steps at temperatures up to 180 °C and lasting up to 7 days. Herein, we demonstrate a significantly streamlined processing regimen, which indicates that much lower temperatures and processing durations can be used without alkaline lysis. By employing CHN elemental analysis, scanning electron microscopy (SEM), confocal laser scanning microscopy (CLSM), and dynamic image particle analysis (DIPA), the optimum conditions for *L. clavatum* SEC processing were determined to include 30 hours acidolysis at 70 °C without alkaline lysis. Extending these findings to proof-of-concept encapsulation studies, we further demonstrate that our SECs are able to achieve a loading of 0.170 ± 0.01 g BSA per 1 g SECs by vacuum-assisted loading. Taken together, our streamlined processing method and corresponding characterization of SECs provides important insights for the development of applications including drug delivery, cosmetics, personal care products, and foods.

Natural capsules obtained from plant spores and pollens are drawing significant attention for diverse microencapsulation applications^1^,^2^,^3^,^4^,^5^,^6^,^7^,^8^,^9^,^10^,^11^,^12^,^13^,^14^,^15^,^16^,^17^. Through various processes^7^,^9^,^18^,^19^,^20^, it is possible to obtain empty exine capsules devoid of cytoplasmic material, proteins, and intine layer^2^,^12^,^21^. The resulting sporopollenin exine capsules (SECs) exhibit a high degree of structural and chemical stability^22^,^23^,^24^,^25^,^26^,^27^, as well as species-specific uniformity with regards to size distribution, morphology, and micromeritic properties, offering an intriguing alternative to existing synthetic encapsulants^7^,^9^,^13^.

Plant spores from the species *Lycopodium clavatum* (Fig. 1A) have become the most widely studied single source of SECs, and have a long history of use as a natural powder lubricant^28^, a base for cosmetics^28^, and in herbal medicine^29^,^30^,^31^,^32^,^33^. It is proposed that this is primarily due to its availability, low cost, and chemical robustness^9^. The exine layer of *L. clavatum* is more resilient to acid and alkali treatment than spores and pollen of many other species^2^. After processing, the resulting SECs retain their intricate microridge structures and high morphological uniformity while providing a large internal cavity for encapsulation^7^ (Fig. 1A). Recent studies of *L. clavatum* SECs as an encapsulant have shown high loading efficiencies with drugs^10^,^13^, vaccines^11^, proteins^7^,^14^, cells^8^, oils^5^,^6^,^7^,^9^, and food supplements^5^,^15^, in comparison to conventional encapsulation materials^7^. There are also reports of *L. clavatum* SEC encapsulation providing taste-masking^6^,^10^ and antioxidant protection^12^.

In this paper, we introduce a systematic analysis of each SEC extraction step for *L. clavatum* spores and show that it is possible to significantly reduce the processing temperatures and durations compared to the most commonly used method. Finally, it was determined that alkaline lysis treatment can be completely removed (Fig. 1B) and still produce SECs of equivalent quality. Further, to demonstrate the functionality of our SECs, they were used for the encapsulation of bovine serum albumin (BSA) as a model system to determine the compound loading efficiency.

## Experimental Section

### Materials

Natural *L. clavatum* spores (S-type)^2^, bovine serum albumin (BSA), FITC-conjugated BSA, and other solvents were purchased from Sigma-Aldrich (Singapore). Phosphoric acid (85% w/v) and hydrochloric acid were procured from Merck (Singapore). Polystyrene microspheres (50 ± 1 μm) were purchased from Thermoscientific (CA, USA). Vectashield (H-1000) medium was purchased from Vector labs (CA, USA) and Sticky-slides, D 263 M Schott glass, No.1.5H (170 μm, 25 mm × 75 mm) unsterile glass slides were purchased from Ibidi GmbH (Munich, Germany). Commercial *Lycopodium* SECs (L-type)^2^ were purchased from Polysciences, Inc. (PA, USA).

### Extraction of Sporopollenin Exine Capsules (SECs)

SECs were extracted by four main chemical processes: defatting, alkaline lysis, acidolysis, and serial washing followed by drying. Natural *L. clavatum* spores (100 g) were suspended in acetone (500 ml) in a round bottomed flask fitted with a glass condenser, and were refluxed at 50 °C for 6 h under gentle stirring. The defatted spores were collected by filtration under vacuum and air dried in a glass dish for 12 h. The dried samples were then refluxed (70 °C) in aqueous 6% (w/v) potassium hydroxide solution (500 ml) with gentle stirring for 6 h. The samples were collected by filtration and washed using MilliQ (MQ) water (2 × 500 ml) before resuming the alkaline lysis for another 6 h using fresh potassium hydroxide solution (500 ml). After the 12 h of alkaline lysis, the SECs were collected by centrifuging at 4500 rpm and washed using hot MQ water (5 × 500 ml) to neutralize the alkaline residues. After each wash the suspension was filtered using vacuum filtration. The SECs were then washed twice using hot ethanol (2 × 500 ml) and dried overnight at 25 °C. The resultant SECs were subjected to acidolysis by suspending capsules in 85% (v/v) phosphoric acid (500 ml) and stirred under gentle reflux at 70 °C for up to 120 h. The phosphoric acid was replenished after 60 h. At each stage of the treatment, 15 ml of the suspension was collected for characterizations. After acidolysis, the SECs were washed by a series of washing steps involving hot water (5 × 800 ml), hot acetone (600 ml), hot 2M hydrochloric acid (600 ml), hot 2M sodium hydroxide (600 ml), hot water (5 × 800 ml), hot acetone (600 ml), and hot ethanol (600 ml), and finally the resulting SECs were collected by vacuum filtration. The washed SECs were transferred to a clean glass dish and air-dried for 12 h. The drying was completed in a vacuum hot plate oven (Memmert, Schwabach, Germany) at 60 °C at 1 mBar for 8 h and the dried SECs were then stored in a dry cabinet until characterization.

### Surface Morphology Evaluation by Scanning Electron Microscopy (SEM)

FESEM 7600F (JEOL, Japan) was used for SEM image processing. Cross section samples were prepared by mounting intact spores or SECs on SEM sample mounting tape, followed by immersion in liquid nitrogen for 30 s. A steel blade (No. 10 round edge carbon steel scalpel blade, RS Components, Singapore) was used to slice across the particles several times. The cut lines were followed during SEM until a suitable cracked particle was found. All samples were coated with platinum at a thickness of 10 nm using JFC-1600 (JEOL, Japan) (20 mA, 60 sec). Images were recorded with an acceleration voltage of 5.00 kV at different magnifications to observe morphological changes before and after each SEC extraction processing step.

### Elemental CHN Analysis

The CHN analysis was carried out with a calibrated VarioEL III elemental analyzer (Elementar, Hanau, Germany). All samples were dried at 60 °C for at least 1 h before elemental analysis and the protein content was calculated using percent nitrogen with a multiplication factor of 6.25^11^. Statistical results were obtained using triplicate measurements for each sample.

### Dynamic Image Particle Analysis (DIPA)

DIPA by FlowCam^®^: The benchtop system (FlowCamVS, Fluid Imaging Technologies, Maine, USA) was equipped with a 200 μm flow cell (FC-200), and a 20X magnification lens (Olympus^®^, Japan). The system was flushed with 1 mL deionized water (Millipore, Singapore) at a flow rate of 0.5 ml/min and flow cell cleanliness was visually inspected before each sample run. Untreated spores, SECs, and BSA-loaded SECs solutions with a concentration of 2 mg/ml were primed manually into the flow cell (a pre-run volume of 0.5 mL) and were analyzed with a flow rate of 0.1 ml/min and a camera rate of 14 frames/s leading to a sampling efficiency of approximately 12.2%. A minimum of 10,000 particles were fixed as the particle count for each measurement and three separate measurements were performed. Data analysis was carried out using ~1000 well-focused particles obtained from the raw data by segregating based on edge gradient. The instrument was calibrated using polystyrene microspheres (50 ± 1 μm) and representative microsphere data is depicted as histograms with Gaussian curves fitted thereto. All specific values are reported with standard deviations (Figure S1).

### Confocal Laser Scanning Microscopy Analysis (CLSM)

CLSM analysis was performed using a Carl Zeiss LSM710 (Germany) confocal microscope equipped with three spectral reflection/fluorescence detection channels, six laser lines (405/458/488/514/561/633 nm), and connected to a Z1 inverted microscope (Carl Zeiss, Germany). Images were collected under the following conditions: laser excitation lines 405 nm (6%), 488 nm (6%), and 561 nm (9%), with DIC using an EC Plan-Neofluar 100 **×** 1.3 oil objective M27 lens. Fluorescence data from untreated spores, SECs, and BSA-loaded SECs was collected in photomultiplier tubes equipped with the following emission filters; 416–477, 498–550, and 572–620. The laser scan speed was set at 67 sec per each phase (1024 **×** 1024: 84.94 μm^2^ sizes) and plane mode scanning was performed with a pixel dwell of 12.6 μs.

### Biomacromolecule Encapsulation by Vacuum Loading Technique

Biomacromolecule loading into SECs was performed using a vacuum loading technique based on a previous protocol^34^. Due to variations in the absorption properties of untreated spores and SECs, 150 mg of untreated spores were suspended in 0.6 ml of 125 mg/ml aqueous BSA solution, and 150 mg SECs were suspended in 1.2 ml of 125 mg/ml aqueous BSA solution in polypropylene tubes to form a homogeneous suspension and then mixed for 10 min using a vortex mixer (IKA, Staufen, Germany). The tubes were transferred to a freeze dryer (Labconco, MO, USA) and a vacuum was applied at 1 mbar for 2 h. The tubes were collected and the loaded SECs were washed using 2 ml water then centrifuged to remove residual BSA, this washing step was repeated for a total of two washes. The SECs were then placed in a freezer at −70 °C for 30 min before freeze-drying for 24 h. The resultant SECs were stored at −20 °C until characterization. Placebo capsules were prepared with the same procedure, except BSA, and also stored at −20 °C. In order to determine the localization of BSA within the SECs, FITC-conjugated BSA was encapsulated in SECs using the same procedure as the above-mentioned vacuum loading technique.

### Encapsulation Efficiency

5 mg of BSA-loaded SECs were suspended in 1.4 mL of PBS, then vortexed for 5 min, and probe sonicated for 3 cycles of 10 s (40% amplitude). The solution was filtered to collect the extracted BSA using a 0.45 μm PES syringe filter (Agilent, CA, USA). The absorbance values were measured at 280 nm (Boeco-S220, Germany) using a placebo extract as a blank and the amount of BSA in the SECs was calculated using a BSA standard curve.

#### Statistical analysis

Statistical analysis was performed using two-tailed *t* –tests and *P* < 0.05 was considered as statistically significant. Quantitative data are reported as mean values ± standard deviation of three separate measurements.

## Results and Discussion

Several *L. clavatum* SEC extraction processes have been developed involving acetolysis, mild enzymatic chemical treatments, or aggressive non-oxidative reagents^4^,^5^,^6^,^7^,^8^,^9^,^10^,^11^,^35^,^36^,^37^. However, acetone defatting, KOH reflux, and phosphoric acid reflux is preferred due to its convenience and utilization of non-toxic and inexpensive reagents^7^.

**Figure 2:**
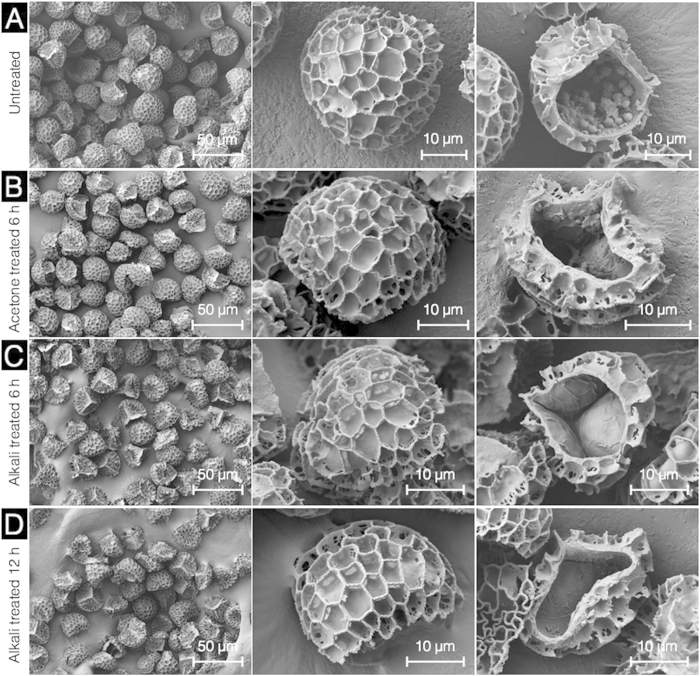
Characterization of *L. clavatum* spores during different stages of pre-acid treatment by scanning electron microscopy. (**A**) Untreated *L. clavatum* spores are intact with uniform size, well defined microstructure, and cross section indicating sporoplasmic cellular organelles and biomolecules. (**B**) Defatted spores at different magnifications are intact and the cross section reveals sporoplasmic biomolecules. (**C**) and (**D**) respectively show SECs after 6 h and 12 h alkaline lysis indicating intact capsules with the cross section revealing residual proteinaceous debris and the cellulosic intine layer.

Conventionally, pre-acid treatement for SEC extraction has involved defatting and alkaline lysis^1^,^2^,^3^,^4^,^5^,^6^,^7^,^8^,^9^,^10^,^11^,^15^. Natural *L. clavatum* spores (Fig. 2A) were characterized with SEM to reveal a uniform morphology and size distribution (31.0 ± 2.2 μm) with reticulate web-like microridges and a tripartite structure as observed in numerous previous studies^4^,^5^,^6^,^7^,^8^,^9^,^10^. The internal cavity of the natural spores appears predominantly hollow with sporoplasmic material forming clusters of cellular organelles and biomolecules^11^. Defatted spores from acetone refluxing exhibit no obvious changes in external spore morphology and microstructures, however, the sporoplasmic contents appeared to have been disrupted and partially removed (Fig. 2B). The effect of alkaline lysis with 6% KOH was examined at both 6 and 12 h. SEM imaging revealed that neither 6 or 12 h alkaline lysis produced obvious changes in the external SEC morphology and microstructures. Nevertheless, the SEM cross-section showed a significant reduction in the sporoplasmic debris. However, on the inner surface of the SEC, there appears a thin (<1 μm) partially wrinkled secondary layer which may be attributed to the cellulosic intine layer^21^. The spore intine layer is reported to be undamaged by alkaline lysis and requires acidolysis to remove^2^,^6^,^7^.

**Figure 3:**
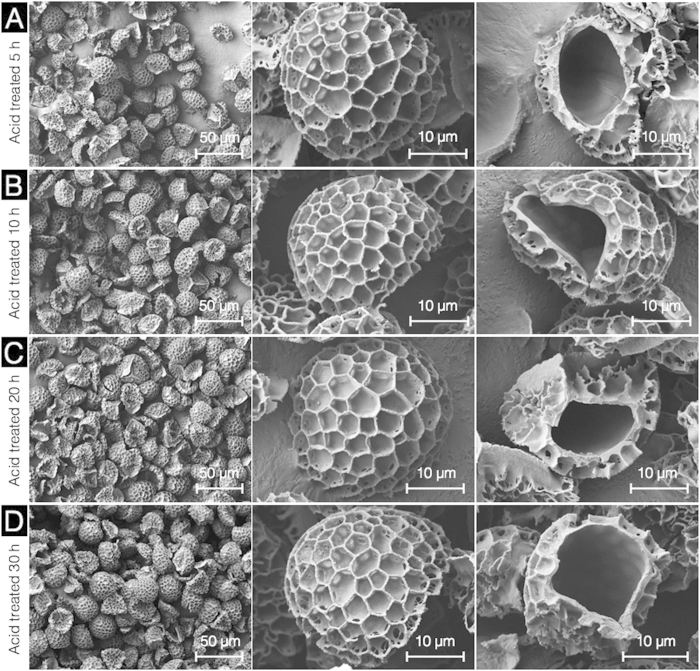
Scanning electron microscopic images of sporopollenin exine capsules (SECs) during different stages of acidolysis using 85% (v/v) phosphoric acid. (**A**) SECs after 5 h acidolysis indicating an intact, well defined microstructure with a clean empty inner cavity. (**B–D**) respectively indicate empty, intact SECs after acidolysis for 10 h, 20 h, and 30 h durations at different magnifications.

Conventionally, acidolysis has been performed with phosphoric acid at temperatures up to 180 °C for up to 7 days (168 h)^1^,^2^,^3^,^4^,^5^,^6^,^7^,^8^,^9^,^10^,^11^,^15^. In order to optimize this process, we first proceeded with SEC acidolysis by phosphoric acid reflux at 70 °C at different time points for up to 120 h. After acid refluxing for 5 to 30 h (Fig. 3), there are no obvious changes in external SEC morphology and microstructures. However, for all of the 5 to 30 h treated SECs, the thin wrinkled intine layer observed after KOH reflux appears to have been removed and the inner surfaces of the capsules are smooth. An acid reflux temperature of 70 °C and duration 5 to 30 h is adequate to produce clean empty SECs which appear to be devoid of the secondary intine layer.

**Figure 4:**
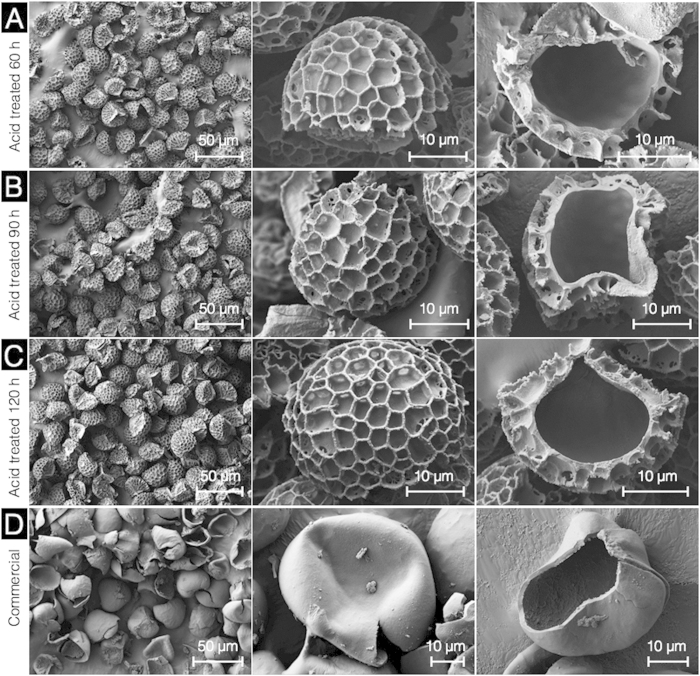
Characterization of sporopollenin exine capsules (SECs) after prolonged acidolysis by scanning electron microscopy. (**A**) SECs after 60 h acidolysis indicating an intact, well defined microstructure with a clean empty inner cavity. (**B**,**C**) respectively indicate SECs after acidolysis for 90 h and 120 h. (**D**) Commercial L-type *Lycopodium* SECs at different magnifications.

The prolonged acidolysis, for 60, 90, and 120 h treatments, resulted in no further visible changes in external or internal SEC morphology and microstructures (Fig. 4). For comparison, SEM images of commercially available (purchased from Polysciences Inc.) *Lycopodium* SECs (L-type), produced by conventional solvent, alkaline, and acid processing methods, indicate heavy damage and significant fragmentation (Fig. 4D).

**Figure 5:**
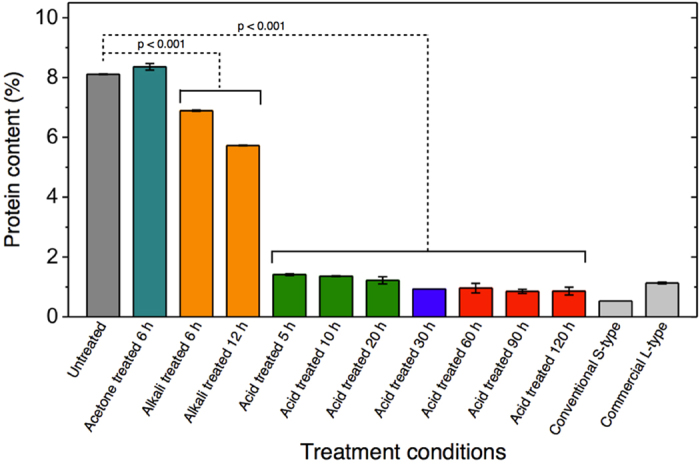
Protein content in sporopollenin exine capsules (SECs) at different stages of treatment. The protein content is obtained from CHN elemental analysis data and is a measure of sporoplasm constituents in untreated spores and treated SECs. Data represented is an average of triplicate measurements with standard deviation (n = 3).

Further, compositional analysis was performed to confirm whether the SECs are adequately devoid of proteins. To compare the protein content of our SECs with those reported in the existing literature, we performed elemental (CHN) analysis of untreated spores and SECs at each stage of our protocol. It is well established that the only major component of plant materials which contain nitrogen is proteins, and that the average nitrogen content of protein in plant materials is considered to be 16%2. Based on this, the protein content of untreated spores was determined to be 8% (Fig. 5), which is in agreement with existing studies^2^,^6^,^7^,^11^.

Acetone refluxing had no significant effect on protein content. Based on statistical analysis of the data (p < 0.001), alkaline lysis resulted in small but statistically significant reductions in protein content, with 6 h and 12 h treatments yielding ~7% and ~6% total protein, respectively. Acidolysis resulted in the largest overall reduction in protein, to less than 2%, even after only the first 5 hours of treatment. Over 10, 20, and 30 h acid reflux, we see a progressive decrease until total protein content reaches ~1%, corresponding to a nitrogen content of 0.15%. Beyond 30 h there is no significant reduction in nitrogen content. Our results of 0.15% nitrogen content are in-line with several well-executed recent studies of SEC encapsulation^2^,^6^,^7^,^11^ as well as commercially available SECs (Table 1). Our data indicates that acidolysis at 70 °C for 30 hours achieves maximum proteinaceous nitrogen removal and that acidolysis is the largest contributor to the removal of proteinaceous nitrogen.

In order to produce high quality SECs for use in microencapsulation and large-scale industrial applications, it is important to confirm the uniformity of SEC micromeritic properties. We have performed a systematic characterization of untreated spores and SECs at each stage of processing using high-throughput dynamic image particle analysis (DIPA) techniques to analyze the micromeritic properties of 1000 particles.

**Figure 6:**
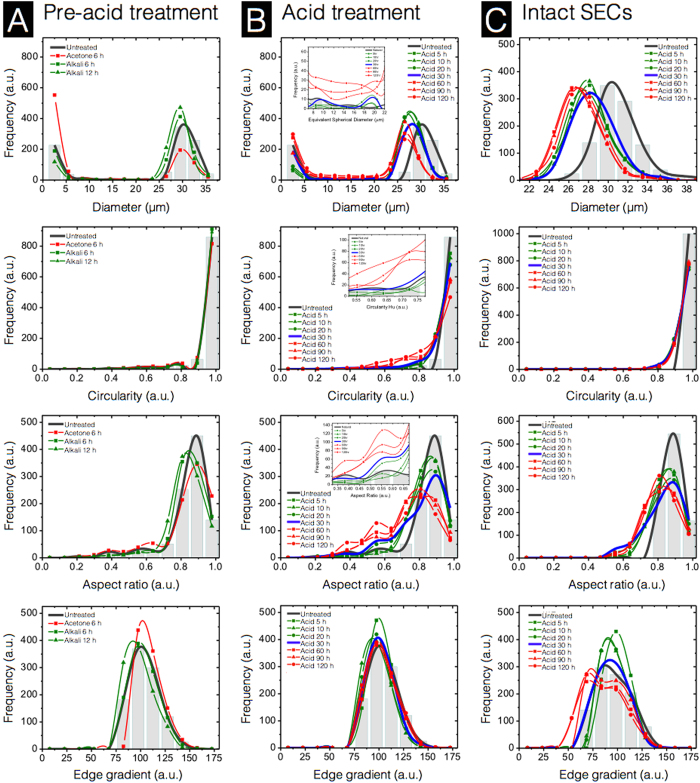
Characterization of sporopollenin exine capsules (SECs) at various stages of treatment using dynamic imaging particle analysis (DIPA). Micromeritic properties of SECs are displayed as column (**A**) Pre-acid treatment, column (B) Acid treatment using 85% (v/v) phosphoric acid at 70 °C, and column (**C**) Intact SECs from 22 to 38 μm in diameter after acidolysis. Plots are representative graphs of diameter, circularity, aspect ratio, and edge gradient, obtained by the spline curve fitting of histogram data from 1000 well-focused particle images after triplicate measurements (n = 3).

Our DIPA data indicates that the average diameter of intact natural untreated *L. clavatum* spores is 31.0 ± 2.2 μm (Fig. 6A). This is in agreement with reported results obtained by laser diffraction, which identified a median diameter of 30.6 μm and a very narrow size distribution^38^. The bimodal distribution observed throughout our SEC diameter data indicates that the majority of the particles in our samples are either intact particles (24–36 μm), or debris (1–7 μm). Natural untreated spores show a small portion of debris, which we attribute to small amounts of surface particulate matter being released during immersion in water during sample preparation.

The defatted spores from acetone reflux show minimal difference in diameter from that of untreated spores, however, defatted pores exhibit a notable increase in the debris population in the 1–7 μm range. Our SEM observations indicate that defatted SECs (Fig. 2B) are intact. Therefore, we attribute the observed increase in debris to additional surface particulate matter being released due to long exposure to acetone. After successive alkaline reflux steps, the portion of 1–7 μm debris returns to a level similar to untreated spores, and continues to decrease with the second alkaline lysis treatment (Fig. 6A). Overall, circularity, and aspect ratio distributions show similar trends and indicate minimal changes in the uniformity of particle morphology throughout the pre-acid phase.

Acid treatment shows a reduction of intact particle diameter from 31.0 ± 2.2 μm, for natural untreated spores, to ~27.5 ± 2.2 μm. The debris population drops significantly for 5, 10, and 20 h treatments but progressively increases from the 30 h treatment. In addition, after 60, 90, and 120 h treatments, there is a significant increase in larger SEC fragments (Fig. 6B, diameter insert) in the diameter range of 7–22 μm. The progressive increase in both the small debris and larger fragments strongly suggests that the structural integrity of the SECs is deteriorating with prolonged acidolysis. The circularity and aspect ratio distributions of the acid treatment data (Fig. 6B) both show clear shifts towards lower values when acidolysis treatment is prolonged beyond 30 h. These shifts indicate decreases in the overall uniformity of particles. In the case of acid-treated intact SECs (Fig. 6C), both of the ~3.5 μm decrease in average particle diameter and the uniformity of intact SEC morphology is shown to be relatively stable (a complete collection of DIPA images of intact SECs are provided in Figure S2 and comparative DIPA data from commercially available SECs is provided in Figure S3). Taken together, SEC structural integrity decreases with prolonged acidolysis and begins to cause significant fragmentation of the SECs beyond 30 h treatment durations.

**Figure 7:**
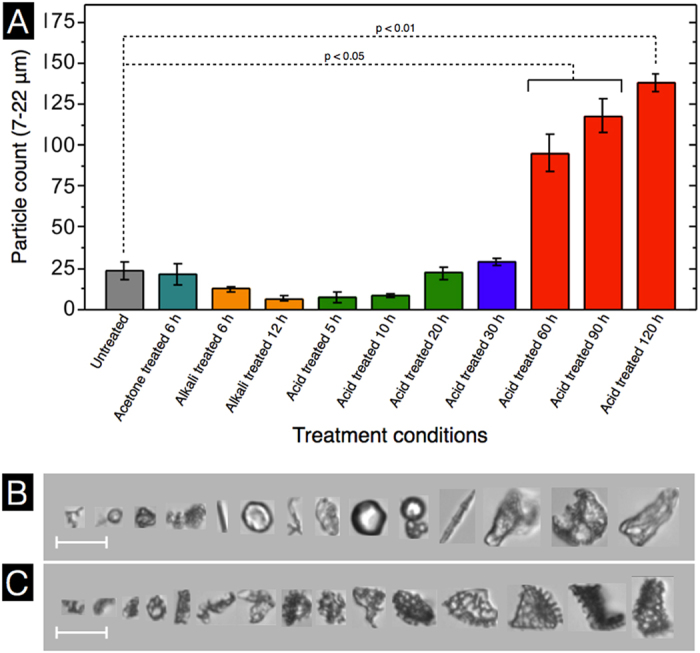
Sporopollenin exine capsule (SEC) fragmentation analysis based on dynamic imaging particle analysis (DIPA) at various stages of processing. (**A**) Fragment count from 1000 well-focused particle images based on particles in the size range of 7 to 22 μm. (**B**) Representative DIPA images of particles in the size range of 7 to 22 μm from natural untreated spores. (**C**) Representative DIPA images of particles in the size range of 7 to 22 μm after 120 h acidolysis treatment. Data represented is an average of triplicate measurements with standard deviation (n = 3). (scale bar is 20 μm).

Fragmentation of SECs was analyzed further by a precise quantification of particles which could be clearly observed to be large portions of the SEC walls. A particle diameter range of 7 to 22 μm was chosen for analysis, as smaller particles may be the result of the web-like reticulum wall (muri) fragmentation only. The natural untreated spores exhibited ~24 fragments (of 1000 particles) (Fig. 7A), which are attributed to plant-based debris (Fig. 7B) rather than spore fragmentation. The number of fragments progressively drops during the pre-acid treatment to less than 10 fragments, and is expected to be due to degradation of the plant-based debris from the natural untreated spores. With acid treatment, a gradual increase to ~29 fragments can be seen within the first 30 h. Prolonged acidolysis results in a substantial jump to ~95 fragments after 60 h and then a steady increase to ~138 fragments (or 13.8% of total particles) after 120 h. A representative collection of SEC fragment images after 120 h treatment is presented in Fig. 7C. These results suggest that SEC structural integrity is progressively reduced with increasing acidolysis treatment times. Therefore, to reduce fracturing and fragmentation, and obtain the highest quality SECs, it is important to minimize the duration of acidolysis. In our study, 30 h appears to be the maximum treatment time permissible to avoid significant reductions in SEC structural integrity.

As an extension of our initial study objectives, we further explored the potential for SEC extraction excluding the use of alkaline solutions. It has been common practice to use alkaline treatments to remove proteins from *L. clavatum* SECs^1^,^2^,^3^,^4^,^5^,^6^,^7^,^8^,^9^,^10^,^11^. However, upon examination of our SEC protein content data (Fig. 5), acid processing demonstrated a much greater effect on total protein removal than the alkali treatment steps. Our data indicates that alkali + phosphoric acid treatment for 30 h produces SECs with optimal characteristics. After alkali +30 h acid treatment, the SEC proteinaceous nitrogen content was reduced to 0.1% and the SEC fragmentation was minimal. Therefore, our next objective was to determine if it is possible to produce SECs of similar quality using defatting and acidolysis only. Three independent 20 g batches of 30 h acidolysis-only SECs were prepared using all the same processing conditions as in our initial study except for the alkaline lysis treatment steps.

**Figure 8:**
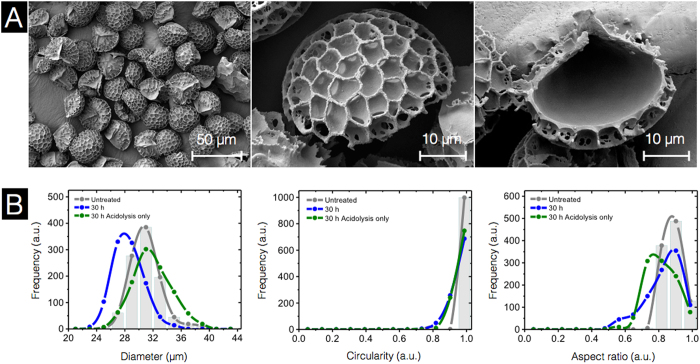
Characterization of 30 h acidolysis-only sporopollenin exine capsules (SECs) using scanning electron microscopy (SEM) and dynamic imaging particle analysis (DIPA). (**A**) SEM images indicating an intact, well defined microstructure with a clean empty inner cavity. (**B**) Micromeritic properties of untreated spores and SECs. Plots are representative graphs of diameter, circularity, and aspect ratio, obtained by the spline curve fitting of histogram data from 1000 well-focused particle images from triplicate independent batches (n = 3).

The SEM results of acidolysis-only SECs indicate no obvious variations in external or internal SEC morphology and microstructures (Fig. 8A) when compared to our previous alkali +30 h acid SECs (Fig. 3D). However, DIPA analysis reveals that the new acidolysis-only SECs have a diameter 1.5 μm larger (32.5 ± 2.7 μm) than untreated spores and 4.0 μm larger than our conventional alkali +30 h acid SECs (Fig. 8B). All other parameters reveal minimal variation in SEC morphology. Overall, the only significant morphological difference between SECs produced with or without alkaline lysis is that acidolysis-only processing produces SECs which are ~4 μm greater in diameter. However, perhaps of most importance, our 30 h acidolysis-only SECs have a residual nitrogen content of only 0.08 ± 0.0%, which is comparable to values discussed above (0.15% for our conventional alkali +30 h acid SECs). Through the removal of the alkaline reflux processing step, the production of SECs becomes substantially more eco-friendly and it becomes more viable to explore industrial-scale applications for this emerging technology.

**Figure 9:**
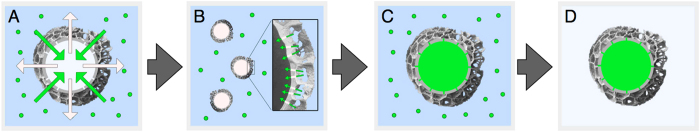
Schematic of microencapsulation of bovine serum albumin (BSA) into intact sporopollenin exine capsules (SECs). (**A**) BSA loading by incubating SECs in aqueous BSA solution under vacuum. (**B**) Uptake of BSA through nanochannels in SEC walls. (**C**) and (**D**) respectively represent an SEC during the loading process and a finally prepared BSA-loaded SEC.

In order to demonstrate the effectiveness of our SECs for use in microencapsulation applications we proceeded with a study to compare the loading efficiency of natural untreated spores with our SECs. To explore the potential encapsulation efficiency of our 30 h acidolysis-only SECs, we chose to use bovine serum albumin (BSA) as a well-studied model protein^39^,^40^,^41^ and encapsulation was achieved utilizing a modified vacuum loading technique^2^,^5^,^6^,^7^. Figure 9 depicts the process of encapsulating BSA within SECs. Briefly, empty SECs are suspended in an aqueous solution of BSA, and with the application of a vacuum, the internal cavity pressure is altered to forcefully draw the BSA solution into the empty capsules (Fig. 9A)^42^ Nanochannels (dia. 15–20 nm), which have been previously identified in the exine walls of *L. clavatum*^43^, allow the solution to enter into the large internal cavity (Fig. 9B,C). After loading, the capsules were washed twice to remove residual BSA from the outer surface, and the BSA-loaded SECs were dried in a freeze-drier to yield a free-flowing powder (Fig. 9D).

Compound loading analysis (Table 2) indicates a 1.30 times greater loading by weight for the 30 h SECs in comparison to untreated spores, with a statistical significance of p < 0.05. The 30 h SECs had a loading of 0.170 ± 0.010 g of BSA per 1 g of SECs, whereas, the natural untreated spores had a loading of 0.131 ± 0.012 g of BSA per 1 g of untreated spores. Percent compound loading and encapsulation efficiency are included in Supplementary Information (Table S1).

**Figure 10:**
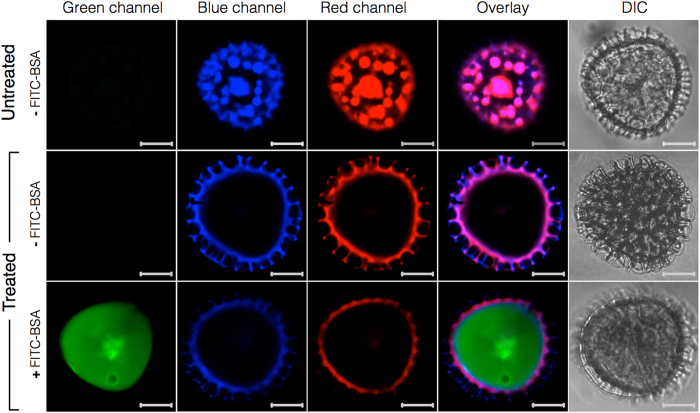
Confocal laser scanning microscopy (CLSM) analysis of sporopollenin exine capsules (SECs) before and after treatment and BSA encapsulation. CLSM images in the first row are unprocessed *L. clavatum* spores indicating autofluorescence due to sporoplasmic cellular organelles and biomolecules. The second row indicates intact SECs after 30 h acidolysis-only treatment with a clean and intact large inner cavity, and the third row indicates FITC-BSA loaded into 30 h acidolysis-only SECs. (Scale bars are 10 μm).

To confirm the cleanliness of the capsules and the effectiveness of encapsulation, we performed confocal laser scanning microscopy (CLSM) for untreated spores as well as 30 h acidolysis-only SECs before and after macromolecule loading (Fig. 10). To observe the localization of loaded BSA, we encapsulated FITC-BSA into 30 h acidolysis-only SECs following the same protocol as mentioned above. FITC fluoresces in the green light spectrum (excitation: 490 nm, emission: 525 nm)^44^ and it is known that plant spores also fluoresce across a broad spectrum of excitation wavelengths^45^,^46^,^47^. To ensure that no overlap was observed in the emission spectrums of the FITC-BSA and untreated spores or SECs, the CLSM settings were calibrated to produce no visible emissions in the green spectrum for either the untreated spores or the 30 h acidolysis-only SECs. After calibration, all scan results shown in Fig. 10 were obtained using the same green spectrum excitation and emission collection settings. The results for untreated spores indicate no autofluorescence in the green channel. However, for the blue and red channels it is clear that the internal spore cavity contains a collection of cellular organelles similar to those observed in our SEM cross section (Fig. 2A) and SEM data from existing studies^11^. It should be noted that the existence of these organelles appears to contradict previous studies^2^,^6^,^7^ wherein CLSM images of *L. clavatum* spores depict that the entire internal cavity of natural spores exhibit autofluorescence. However, this observation may be due to species-specific variations dependent on material source. The 30 h acidolysis-only SECs show a similar morphology to untreated spores, but with an empty internal cavity. After loading with FITC-BSA and washing to remove residual surface adhered BSA, the SECs exhibit near complete loading of the internal cavity and no binding of BSA to the reticulate ridge structures of the SEC surface. It is important to acknowledge that during the loading of many common encapsulation materials, some portion of the loaded compound may bind to the external surface of the encapsulant^39^,^40^,^41^. However, our CLSM data depicts no surface binding of BSA from the encapsulation process utilized in this study. A series of Z-stack CLSM images for FITC-BSA loaded 30 h acidolysis-only SECs is included in Supplementary Information (Figure S4).

**Figure 11:**
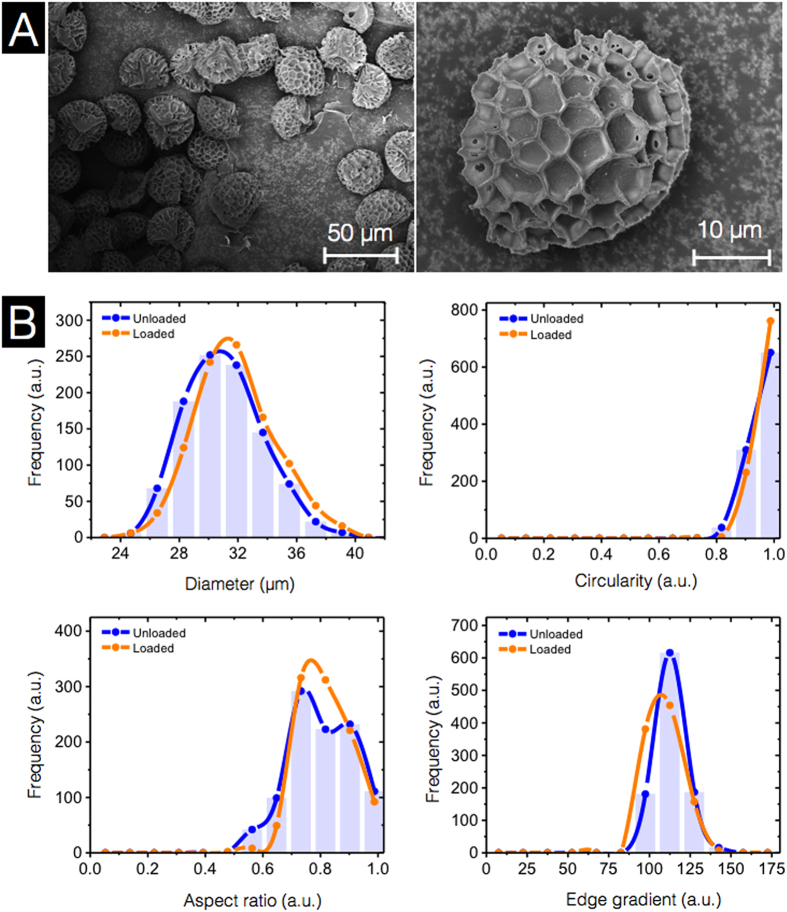
Characterization of BSA-loaded sporopollenin exine capsules (SECs) using scanning electron microscopy (SEM) and dynamic imaging particle analysis (DIPA). (**A**) SEM images of BSA-loaded 30 h acidolysis-only SECs. (**B**) Micromeritic properties of BSA-loaded 30 h acidolysis-only SECs. Plots are representative graphs of diameter, circularity, aspect ratio, and edge gradient, obtained by the spline curve fitting of histogram data from 1000 well-focused particle images from triplicate independent batches (n = 3).

Size uniformity and morphological characteristics are important factors for evaluating the quality of a successful encapsulant. Therefore, we utilized SEM and DIPA to investigate the stability of SEC micromeritics throughout the loading process. From the SEM images in Fig. 11A, the loaded SECs exhibit no obvious changes in morphology. Inspection of the single loaded SEC reveals no BSA binding to the microridge surface structure. This is in agreement with the CLSM observation discussed above. The DIPA data indicates that there are no significant changes in morphology before and after loading with respect to diameter, circularity, aspect ratio, and edge gradient (Fig. 11B). Overall, the process of the vacuum loading of BSA used in our study has had no influence on the uniformity of SEC micromeritic properties.

## Conclusions

We have demonstrated that it is possible to produce higher quality SECs from *L. clavatum* spores while also achieving a significant streamlining of the conventional extraction protocol. By reducing the acidolysis time to 30 h and reflux temperature to 70 °C, as well as the complete removal of the alkaline lysis step, the environmental impact of SEC processing can be reduced and SECs with higher structural integrity are obtained. The extracted SECs retain the well-defined microstructure of natural untreated spores with effective removal of cytoplasmic and cellulosic contents. Importantly, the analysis of the micromeritic properties of large samples of SECs, at various stages of chemical treatment, highlights the detrimental effect of prolonged acidolysis and indicates progressive reductions in structural integrity in the form of increased SEC fragmentation. Compound loading by simple loading techniques, goes on to highlight the versatile nature of these capsules for microencapsulation applications. Taken together, these results are important for the large-scale industrial production of SECs and for stimulating greater interest in the potential applications of this field of research. We envision that the systematic study of SEC extraction from *L. clavatum* spores will lead the way for further exploration of new SECs extracted from a wide variety of plant species.

## Additional Information

**How to cite this article**: Mundargi, R. C. *et al*. Eco-friendly streamlined process for sporopollenin exine capsule extraction. *Sci. Rep.* **6**, 19960; doi: 10.1038/srep19960 (2016).

## Supplementary Material

Supplementary Information

## Figures and Tables

**Figure 1:**
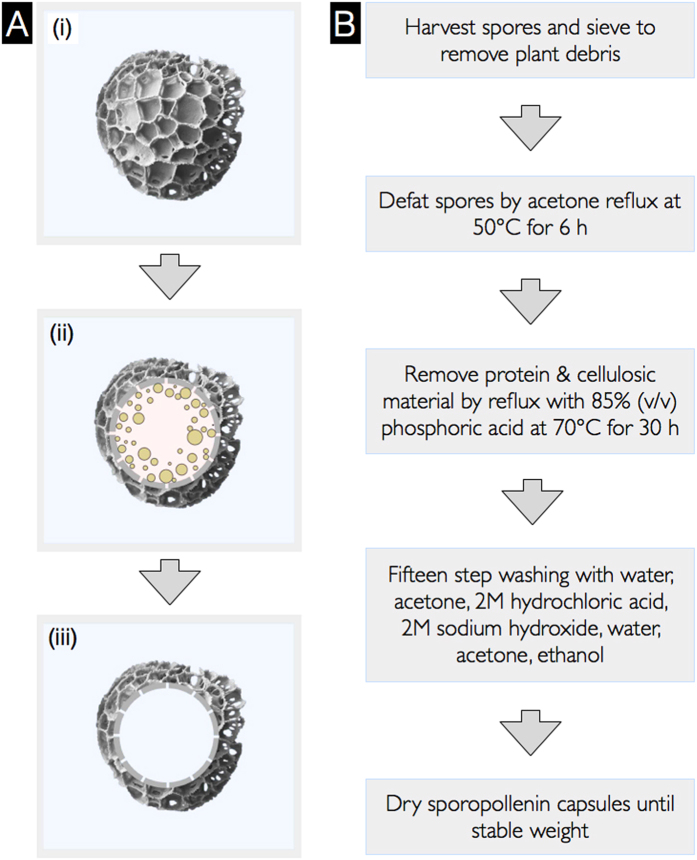
Process of extracting sporopollenin exine capsules (SECs) from spores. (**A**) Schematic of plant spores and SECs, (i) Untreated *L. clavatum* spore, (ii) Spores containing sporoplasmic organelles, and (iii) SEC after removal of sporoplasmic organelles and biomolecules. (**B**) Flowchart of more eco-friendly processes to translate plant spores into SECs.

**Table 1:** Sporopollenin exine capsules (SECs): CHN composition(a).

Treatment conditions	Carbon (%)	Hydrogen (%)	Nitrogen (%)
Untreated	65.4 ± 0.3	9.5 ± 0.1	1.30 ± 0.0
Acetone-6 h	65.6 ± 0.1	9.3 ± 0.0	1.33 ± 0.0
Alkaline lysis-6 h	62.8 ± 0.3	8.9 ± 0.0	1.10 ± 0.0
Alkaline lysis-12 h	62.5 ± 0.1	8.9 ± 0.0	0.91 ± 0.0
Acidolysis-5 h	61.5 ± 0.2	8.2 ± 0.0	0.22 ± 0.0
Acidolysis-10 h	61.8 ± 0.1	8.1 ± 0.0	0.21 ± 0.0
Acidolysis-20 h	62.9 ± 0.3	8.4 ± 0.0	0.19 ± 0.0
Acidolysis-30 h	63.6 ± 0.1	8.6 ± 0.0	0.15 ± 0.0
Acidolysis-60 h	62.1 ± 0.2	8.0 ± 0.1	0.15 ± 0.0
Acidolysis-90 h	62.1 ± 0.6	8.0 ± 0.1	0.13 ± 0.0
Acidolysis-120 h	61.1 ± 1.4	8.2 ± 0.1	0.13 ± 0.0
Commercial	65.6 ± 0.3	6.1 ± 0.1	0.18 ± 0.0

^(a)^CHN analysis performed in triplicate and reported as average values with standard deviation.

**Table 2:** Encapsulation of Bovine Serum Albumin into Untreated Spores and SECs.

**Material**	**BSA loading per batch**(a) **(125** **mg/ml)**	**BSA in 5** **mg SECs (mg)**	**Amount of BSA loaded (g per g of SECs)**
Untreated spores	0.6 ml	0.654 ± 0.05	0.131 ± 0.01
SECs	1.2 ml	0.831 ± 0.05	0.170 ± 0.01

^(a)^Volume of BSA solution used for loading per 150 mg batch of ‘untreated spores’ or ‘SECs’.
