# Supplementary material for: Eco-friendly streamlined process for sporopollenin exine capsule extraction
Source: Sci Rep. 2016 Jan 28;6:19960. doi: 10.1038/srep19960 (PMC4730194; doi:10.1038/srep19960)
Supplement: Supplementary Information [file srep19960-s1.pdf]

Supplementary information

Eco-friendly streamlined process for sporopollenin exine capsule extraction

Raghavendra C. Mundargi, Michael G. Potroz, Jae Hyeon Park, Jeongeun Seo, Ee-Lin. Tan, Jae Ho Lee, and Nam-Joon Cho

Figure S1. Dynamic imaging particle analysis (DIPA): Polystyrene microspheres as a standard (50 ± 1 µm) (Thermoscientific, USA). Plots are representative graphs of (A) diameter, (B) circularity, (C) aspect ratio, and (D) edge gradient, obtained by the spline curve fitting of histogram data from 1000 well-focused particle images after triplicate measurements (n = 3). (E) Representative microsphere images from DIPA.

Figure S2. Representative DIPA images of intact sporopollenin exine capsules (SECs) at various stages of treatment.

Figure S3. Dynamic imaging particle analysis (DIPA): Commercial sporopollenin exine capsules (SECs). Plots are representative graphs of (A) diameter, (B) circularity, (C) aspect ratio, and (D) edge gradient, obtained by the spline curve fitting of histogram data from 1000 well-focused particle images after triplicate measurements (n = 3). (E) Representative SEC images from DIPA.

Table S1. Compound Loading and Encapsulation Efficiency of Bovine Serum Albumin into Untreated Spores and SECs.

Figure S4. Confocal laser scanning microscopy (CLSM) Z-stack analysis of 30 h acidolysis-only SECs loaded with FITC-BSA.

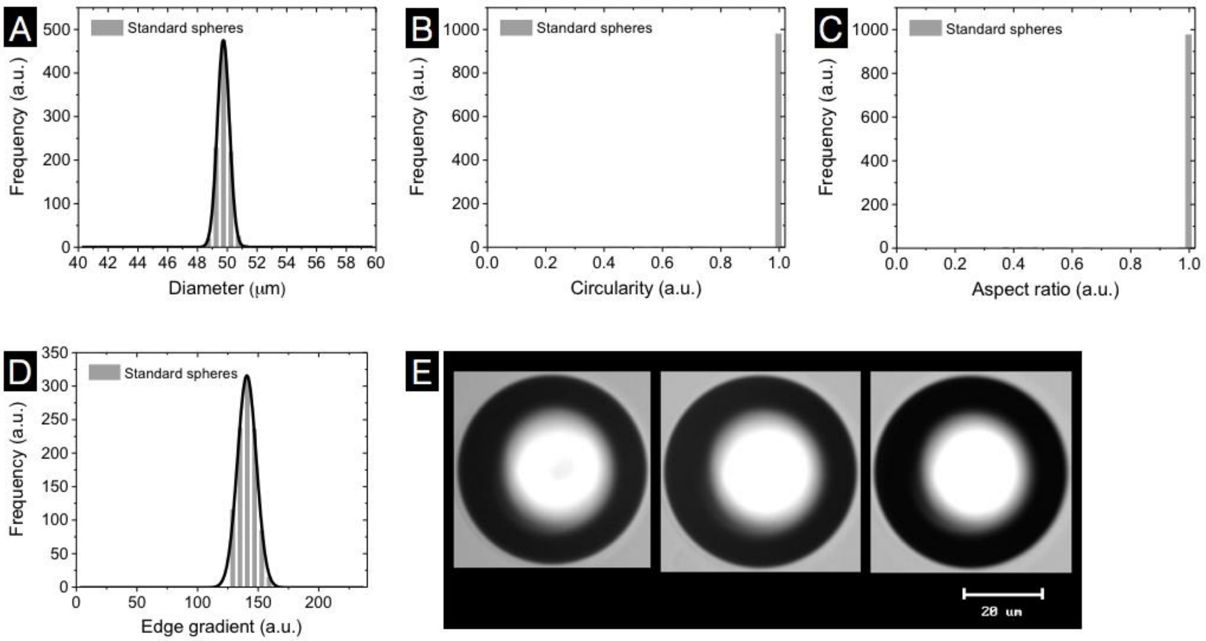

Figure S1. Dynamic imaging particle analysis (DIPA): Polystyrene microspheres as a standard (50 ± 1 μm) (Thermoscientific, USA). Plots are representative graphs of (A) diameter, (B) circularity, (C) aspect ratio, and (D) edge gradient, obtained by the spline curve fitting of histogram data from 1000 well-focused particle images after triplicate measurements (n=3). (E) Representative microsphere images from DIPA.

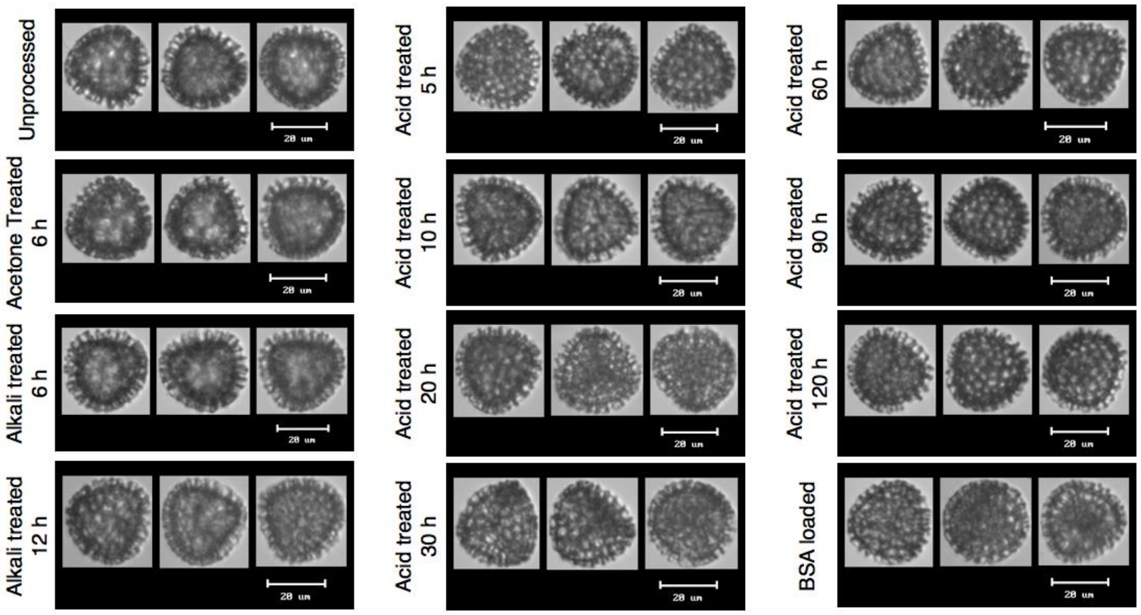

Figure S2. Representative DIPA images of intact sporopollenin exine capsules (SECs) at various stages of treatment.

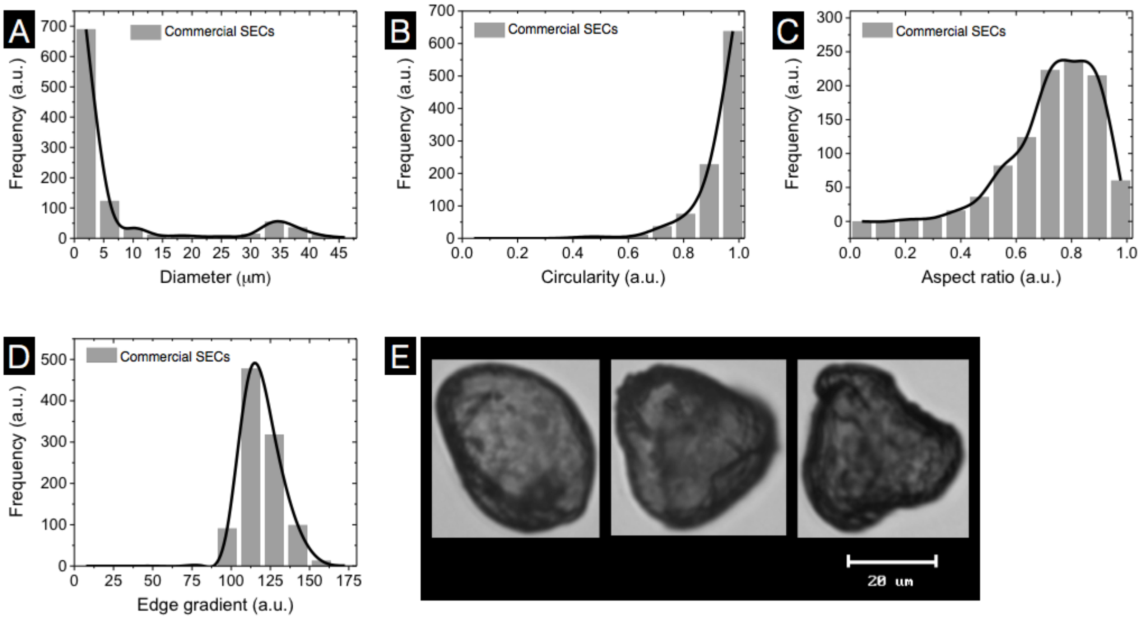

Figure S3. Dynamic imaging particle analysis (DIPA): Commercial sporopollenin exine capsules (SECs). Plots are representative graphs of (A) diameter, (B) circularity, (C) aspect ratio, and (D) edge gradient, obtained by the spline curve fitting of histogram data from 1000 well-focused particle images after triplicate measurements (n=3). (E) Representative SEC images from DIPA.

Table S1. Compound Loading and Encapsulation Efficiency of Bovine Serum Albumin into Untreated Spores and SECs.

	Untreated spores	SECs
% Compound loading	13.6 ± 1.1	26.1 ± 2.3
% Encapsulation efficiency	16.6 ± 1.0	16.6 ± 1.0

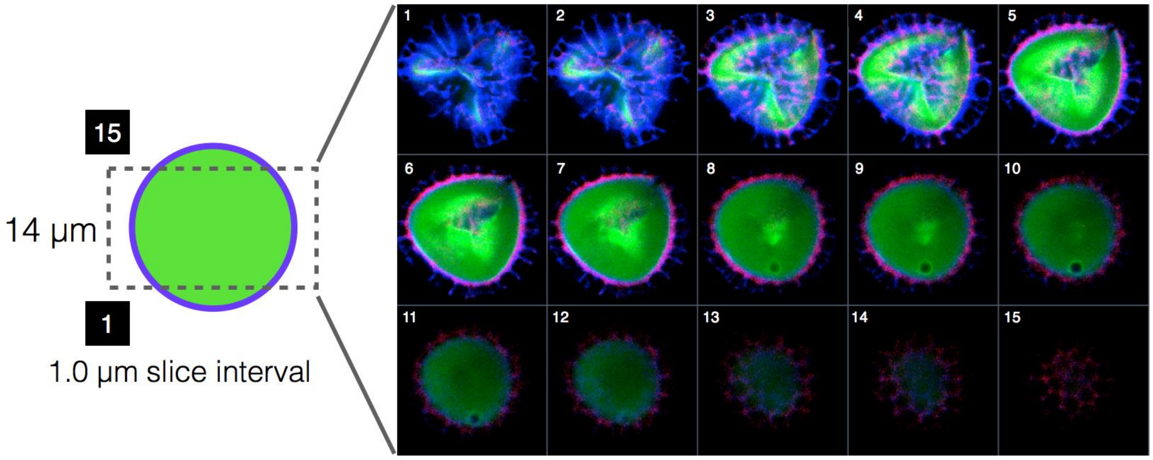

Figure S4. Confocal laser scanning microscopy (CLSM) Z-stack analysis of 30 h acidolysis-only SECs loaded with FITC-BSA.
